# Cell Attachment and Spreading on Carbon Nanotubes Is Facilitated by Integrin Binding

**DOI:** 10.3389/fbioe.2018.00129

**Published:** 2018-09-24

**Authors:** Mozhdeh Imaninezhad, Joseph Schober, David Griggs, Peter Ruminski, Irma Kuljanishvili, Silviya Petrova Zustiak

**Affiliations:** ^1^Biomedical Engineering, Saint Louis University, Saint Louis, MO, United States; ^2^Pharmaceutical Sciences, Southern Illinois University, Edwardsville, IL, United States; ^3^Molecular Microbiology & Immunology, Saint Louis University, Saint Louis, MO, United States; ^4^Center for World Health and Medicine, Saint Louis University, Saint Louis, MO, United States; ^5^Physics, Saint Louis University, Saint Louis, MO, United States

**Keywords:** carbon nanotubes, hydrogel, cell spreading, integrins, protein adsorption, fibronectin

## Abstract

Owing to their exceptional physical, chemical, and mechanical properties, carbon nanotubes (CNTs) have been extensively studied for their effect on cellular behaviors. However, little is known about the process by which cells attach and spread on CNTs and the process for cell attachment and spreading on individual single-walled CNTs has not been studied. Cell adhesion and spreading is essential for cell communication and regulation and the mechanical interaction between cells and the underlying substrate can influence and control cell behavior and function. A limited number of studies have described different adhesion mechanisms, such as cellular process entanglements with multi-walled CNT aggregates or adhesion due to adsorption of serum proteins onto the nanotubes. Here, we hypothesized that cell attachment and spreading to both individual single-walled CNTs and multi-walled CNT aggregates is governed by the same mechanism. Specifically, we suggest that cell attachment and spreading on nanotubes is integrin-dependent and is facilitated by the adsorption of serum and cell-secreted adhesive proteins to the nanotubes.

**Graphical Abstract:**
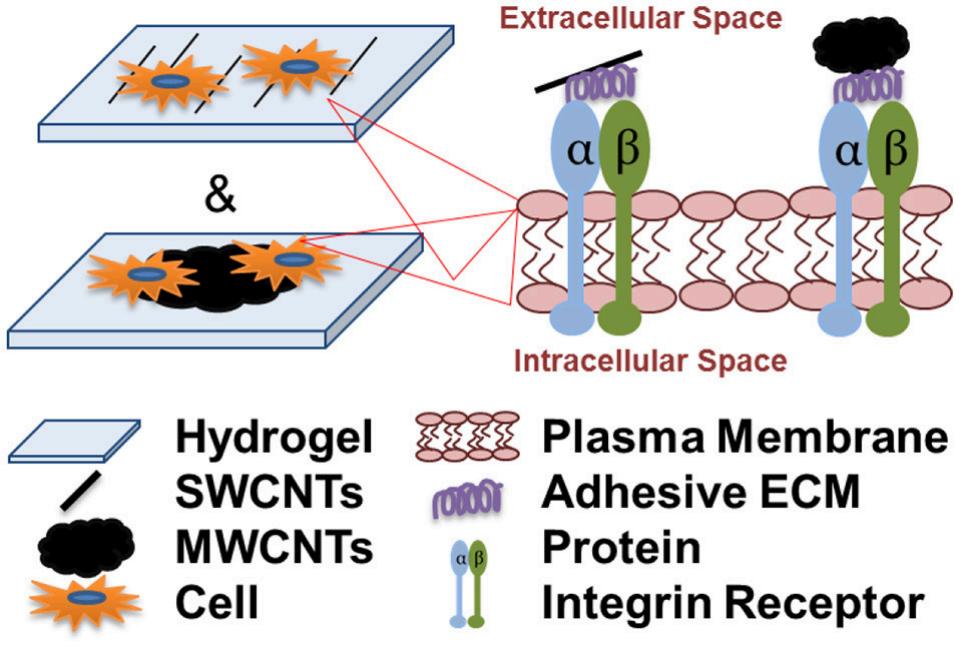
Cell attachment and spreading onto individual single-walled carbon nanotubes (SWCNTs) and multi-walled CNT aggregates (MWCNTs) is integrin-dependent and is facilitated by the adsorption of serum and cell-secreted adhesive extracellular matrix (ECM) proteins to the nanotubes.

## Introduction

Carbon nanotubes (CNTs) are cylindrical and have nanometer diameters but typically micrometer lengths, resulting in high aspect ratios. Owing to their exceptional physical, chemical, and mechanical properties, they have been extensively studied for their effect on cellular behaviors (Ryoo et al., 2010; Bosi et al., 2013). Accounts of tissue engineered products including CNTs have proliferated in recent years, which could be coupled to our growing awareness of the nano-dimensionality of nature (Harrison and Atala, 2007; Shah et al., 2015). In addition, due to their electro-conductivity, CNTs have been used as components of engineered excitable tissues for neural (Lewitus et al., 2011; Bosi et al., 2013) and cardiac (Shin et al., 2013) tissue engineering applications. Due to their exceptional mechanical stability and low weight-to-strength ratio, they have also been used in orthopedic applications (Sahithi et al., 2010), where CNT-based nanofibers have even been shown to select for osteoblast adhesion (Price et al., 2004).

However, very little is known about the mechanism of cell attachment and spreading on CNTs. In one study of soluble multi-walled (MWCNTs) toxicity toward A549 lung cancer cells, the authors observed that some of the MWCNTs coated the surface of the cells (Kaiser et al., 2013). The authors suggested that cells could be adhering directly to the MWCNTs *via* integrins or to serum proteins which have adsorbed onto the MWCNTs first (Kaiser et al., 2013). In another study of neural cells onto MWCNTs aggregates, it was suggested that cells adhered *via* physical entanglements of cellular processes with MWCNT aggregates but only when both were of similar dimensions (Sorkin et al., 2009). However, another recent study of mesenchymal stem cells (MSCs) has shown that cells can recognize, adhere to and spread along individual single-walled (SWCNTs) of a diameter <2 nm; non-specific cell adhesion was controlled through PEG passivation (Namgung et al., 2011). While the mechanism of cell attachment and spreading was not studied, the authors showed that cells formed robust focal adhesion complexes on the SWCNT patterned substrates (Namgung et al., 2011). A different group has shown that NIH 3T3 cells not only formed focal adhesion complexes when grown on MWCNTs films, but the complexes were larger in number and smaller in area compared to a glass substrate, suggesting high affinity for the MWCNTs (Ryoo et al., 2010). Lastly, research on a non-adhesive SiO_2_ substrate has shown that topography alone can contribute to cell adhesion (Fan et al., 2002) and nanometer surface roughness has been shown to increase osteoblast adhesion to carbon nanofibers (Price et al., 2004).

Cell adhesion and spreading is essential for cell communication and regulation and the mechanical interaction between cells and the underlying substrate can influence and control cell behavior and function (Geiger et al., 2001). These interactions play an integral role in the development and maintenance of tissues (Huang et al., 2003). Due to its significance, mechanisms of cell attachment and spreading have been widely explored in various fields such as cellular biology (Kwon et al., 2007) or biomedical applications (Wang et al., 2009). *In vitro* most mammalian cells are anchorage-dependent and attach firmly to the substrate (Sagvolden et al., 1999). Upon cell adhesion, cells undergo morphologic alteration driven by passive deformation and active reorganization of the cytoskeleton. Integrin receptors and heterodimeric transmembrane proteins play a central role in cell adhesion and spreading. For example, fibroblast cells adhesiveness to fibronectin is reduced by impairing α5β1 integrin (Zou et al., 2002). Specific integrin binding provides not only a mechanical linkage between the intercellular actin cytoskeleton and the extracellular matrix, but also a bidirectional transmembrane signaling pathway (Hynes, 1987; Geiger et al., 2001; Van der Flier and Sonnenberg, 2001). Hence, cell adhesion and spreading on the underlying substrate is an important consideration in biomaterial design and development. Further, the requirements for cell adhesion and spreading will differ for different applications and could also be cell-specific (Huang et al., 2003). Surface properties of materials also influence the composition of the adsorbed protein layers, which subsequently regulate a variety of cell behaviors such as attachment, viability, spreading, migration, and differentiation (Webb et al., 2000).

To date there have been very few and contradicting reports on the mechanism of cell attachment and spreading on CNTs, hence no consensus has been reached. Importantly, the mechanism of cell attachment and spreading to individual SWCNTs has not been studied. Here, we hypothesized that cell attachment and spreading to both individual SWCNTs and MWCNT aggregates is governed by the same process. Specifically, we suggest that cell attachment and spreading onto nanotubes is integrin-dependent and is facilitated by the adsorption of serum and cell-secreted adhesive proteins to the nanotubes.

## Materials and methods

### Materials

Single crystal ST cut quartz wafers (diameter of 76.2 mm, thickness of 500 μm) were purchased from University Wafer (Boston, MA). Poly(ethylene glycol) diacrylate (PEGDA, MW 5 kDa) was purchased from Laysan Bio (Arab, AL). Phosphate buffered saline (PBS, 10X, pH 7.4), Hoechst 33258, Alexa 488 phalloidin, bovine serum albumin (BSA) and bovine fibronectin were purchased from Thermo Scientific (Waltham, MA). Rabbit anti-fibronectin antibody was purchased from Abcam (Cambridge, MA). Goat anti-rabbit IgG conjugated with TRITC was purchased from Jackson ImmunoResearch Inc. (West Grove, PA). Irgacure^Ⓡ^ 2959 was purchased from BASF Corporation (Florham Park, NJ). GelBond (GelBond^Ⓡ^ PAG film for polyacrylamide) was purchased from GE Health Care (Filial Sverige, Sweden). Silicone spacers were purchased from Grace Bio-Labs (Bend, Oregon). RainX were purchased from a general store. Ham's F12K medium with 2 mM glutamine and 1.5 g/L sodium bicarbonate, penicillin/streptomycin (pen/strep), and fetal bovine serum (FBS) were purchased from Hyclone (Logan, UT). Dulbecco's modified eagle's medium (DMEM) and N2 supplement (100X) were purchased from Gibco™ (Logan, UT). NIH 3T3 cells were purchased from ATCC (Manassas, Virginia). PC12 cells were generously provided by Dr. Grant Kolar (Department of Pathology, Saint Louis University). 18 × 18 mm #2 glass coverslips and PBS, without calcium and magnesium were purchased from Corning Life Sciences (Manassas, VA). Triton X-100 and formaldehyde were purchased from Sigma Aldrich (St. Louis, MO). Peptidomimetics CWHM-12 and CWHM-96 were synthesized and generously provided by Drs. Griggs and Ruminski (Saint Louis University) (Henderson et al., 2013). A sample of semiconducting multi-walled carbon nanotube powder (MWCNTs) (20 ± 3 nm in diameter and 3 ± 2 μm in length) produced via catalytic chemical vapor deposition was generously provided from MerCorp (Tucson, AZ).

### 4-arm PEG-Ac modification with RGDS

4-arm PEG-RGDS with 80% modification efficiency was prepared following a previously developed protocol (Zustiak and Leach, 2010). Briefly, Glycine-Arginine-Cysteine-Aspartic Acid-Arginine-Glycine-Aspartic Acid-Serine (GRCD-RGDS) was dissolved in 5% acetic acid in de-ionized (DI) water and 4-arm PEG-Ac was dissolved in 0.3 M TEA buffer. The two solutions were combined at a 1:4 molar ratio of RGDS:Ac and reacted for 30 min at room temperature. The concentrations were chosen such that, assuming an ideal reaction, one of the acrylate groups of each PEG-Ac can be conjugated with RGDS. Upon reaction the solution was placed at −80°C for 15 min and then lyophilized (VirTis Sentry 2.0 Lyphilizer) overnight. The 4-arm PEG-RGDS product was purged with argon and stored in a desiccated environment at −20°C until use.

### Polyethylene glycol hydrogel preparation

Stock solution of 1% w/v Irgacure 2959 was prepared by dissolving Irgacure 2959 in DI water and sonicating it in a bath sonicator (Fisher Scientific, Waltham, MA) for 1 h. The Irgacure solution was stored shielded from light at 4°C until use. Stock solution of 30% w/v PEGDA was prepared in 1X PBS, pH 7.4 and stored at 4°C for up to 1 wk. Then, 10% and 20% w/v PEGDA hydrogel precursor solutions containing 0.1% w/v Irgacure 2959 were prepared by diluting the PEGDA and Irgacure stock solutions with 1X PBS pH 7.4 and vortexing for 30 s. To make PEG-RGDS gels, 1% w/v of functionalized 4-arm PEG-RGDS was added to the PEGDA precursor solution (the total PEG concentration was kept at 10 or 20% w/v for all gels). To prepare hydrogels in slab geometry, 50 μl of a hydrogel precursor solution was deposited on a glass plate, which was hydrophobic-coated with RainX. The hydrogel precursor solution was then sandwiched with a second glass plate, where the two plates were separated by silicon spacers (0.5 mm in height). Hydrogels were formed by exposure to 365 nm ultraviolet (UV) light (4.81 mW/cm^2^) for 10 min.

### Cell culture and maintenance

PC12 cells were cultured in Ham's F12K medium with 2 mM L-glutamine and 1.5 g/L sodium bicarbonate, supplemented with 10% FBS, and 1% pen/strep in a humidified environment at 37°C and 5% CO_2_. NIH 3T3 cells were cultured in DMEM medium supplemented with 10% FBS and 1% pen/strep in a humidified environment at 37°C and 5% CO_2_. For cell studies in “serum-free medium,” cells were pre-conditioned for 24 h with DMEM medium supplemented with 1X N2 supplement and 1% pen/strep. Follow up experiments were conducted in a serum-free medium of the same composition. For cell studies in “conditioned serum-free medium,” cells were pre-conditioned for 24 h with DMEM medium supplemented with 1X N2 supplement and 1% pen/strep. This “conditioned serum-free medium,” which was expected to contain soluble cell-secreted adhesive proteins such as fibronectin, was then collected and used in further experiments. For cell maintenance, medium was replaced every 2–3 days until cell confluency was reached. To subculture, confluent cells were harvested by a 5 min exposure to 0.05% trypsin/0.02% EDTA.

### SWCNT transfer from quartz wafers onto hydrogels (SWCNTs/hydrogel)

**Figure 1:**
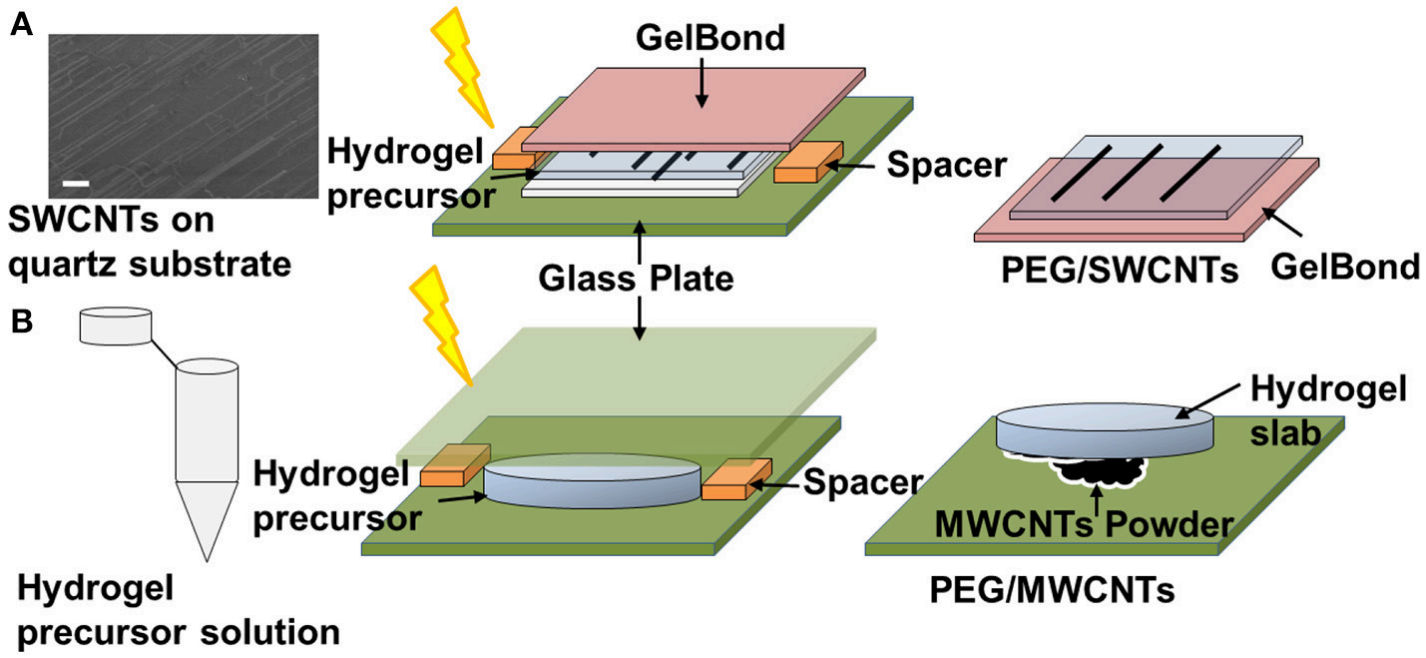
Schematic of CNT-hydrogel nanocomposites preparation. **(A)** Single-walled carbon nanotubes (SWCNTs) are grown on a quartz wafer in a first step. Representative SEM image shows the actual SWCNTs (scale bar is 10 μm). Then a hydrogel precursor solution is deposited on the quartz-grown SWCNTs, sandwiched with a GelBond flexible plastic support and exposed to 365 nm ultraviolet (UV) light (4.81 mW/cm^2^) for 10 min. Lastly, the GelBond/hydrogel construct is peeled off from the wafer with SWCNTS transferred onto the hydrogel. This nanocomposite is termed PEG/SWCNTs. **(B)** A hydrogel slab is made in a first step and then “stamped” onto dispersed multi-walled carbon nanotubes (MWCNTs) to form PEG/MWCNTs nanocomposites.

Figure 1A illustrates the developed technique for transferring quartz-grown single walled carbon nanotubes (SWCNTs) onto a hydrogel substrate, which was previously reported (Imaninezhad et al., 2017). Briefly, aligned SWCNTs were grown on a quartz wafer via a catalytic vapor deposition as described by us previously (Imaninezhad et al., 2017). To transfer the SWCNTs from the quartz wafer onto the hydrogel, 20 μl of the hydrogel precursor solution was deposited directly on the wafer. The hydrogel precursor solution was then sandwiched with a GelBond film, hydrophilic side-down. GelBond is a transparent, flexible film that has a hydrophilic and a hydrophobic side, where the hydrophilic side adheres to the hydrogel during gelation and facilitates easy hydrogel removal from the quartz. Upon gelation, the gel sandwich was soaked in DI water for 2 h to allow for hydrogel swelling and to dissolve remaining salts and Irgacure 2959. Finally, the GelBond/hydrogel was peeled off from the quartz wafer with transferred SWCNTs on its surface.

### Stamped MWCNTs on PEG hydrogels (MWCNTs/hydrogel)

To prepare stamped MWCNTs on PEGDA hydrogels (Figure 1B), first, PEGDA hydrogels were made as described above. Then MWCNTs powder was dispersed on a glass plate and the PEGDA gel slabs were pressed firmly against the MWCNT powder to embed the nanotubes into the hydrogel. The MWCNTs stamped gels were then gently rinsed with PBS to remove loose MWCNTs.

### Scanning electron microscopy imaging of SWCNTs on quartz wafers

SWCNTs were imaged by scanning electron microscopy (SEM, Zeiss EVO LS15 SEM, Oberkochen, Germany). Images were acquired with low acceleration voltages of 1-1.5 kV at various magnifications under a high vacuum environment.

### Evaluation of cell area and shape

To test the ability of SWCNT/hydrogel and MWCNT/hydrogel composites to support cell adhesion and spreading, hydrogels were prepared as described above with and without CNTs. For the control samples without CNTs, PEG hydrogels containing immobilized RGDS (0.08 mM) were prepared to elicit cell attachment. All hydrogel samples (prepared in the form of slabs) were placed at the bottom of 24 well-plates, one sample per well and were sterilized under UV (302 nm) for 60 min. Then, 50 μl of cell suspension was carefully pipetted on top of each hydrogel to achieve a density of 5 × 10^4^ cells/cm^2^ and the plates were placed in an incubated environment at 37°C and 5% CO_2_ for 30 min to initiate cell attachment. Then 500 μl of additional medium was added carefully to each sample and the cells were cultured for up to 24 h. At 24 h cells were washed gently with PBS, covered again with fresh medium, and imaged under an inverted fluorescent microscope (Zeiss, Axiovert 200M, Oberkochen, Germany) at 10X zoom. All images were analyzed with ImageJ software available freely from the NIH at http://rsbweb.nih.gov/ij/. Shape factor was calculated as:

(1) $$\begin{array}{l} f=\frac{4\pi A}{P^{2}} \end{array}$$

where, *A* is cell area and *P* is cell perimeter. A shape factor close to 1 indicates circularity and a shape factor close to 0 indicates elongation.

### Immunofluorescence for fibronectin detection

Fibronectin was passively adsorbed to the PEG, MWCNTs/hydrogel and SWCNTs/hydrogel samples. Fibronectin was diluted to 50 μg/ml in 1X PBS and 10 μl of the diluted solution was deposited on each sample and placed in the incubator for 1 h at 37°C and 5% CO_2_. The samples were rinsed with 1X PBS and then 50 μl cell suspension of NIH 3T3 cells was seeded onto each sample to give a cell seeding density of 5 × 10^4^ cells/cm^2^. Samples were placed in a humidified incubator at 37°C and 5% CO_2_ for 30 min to allow initial cell attachment and then 500 μl of fresh medium was added to each sample for further culturing. At specified time points, the samples were fixed in 4% formaldehyde and 0.1% Triton X-100. After fixation, samples were washed in DI water, and blocked for 20 min at 22°C with 2% w/v BSA solubilized in PBS. Samples were incubated with Alexa 488 phalloidin, Hoechst 33258, and rabbit anti-fibronectin followed by anti-rabbit TRITC secondary antibody, and then washed, and mounted onto standard glass slides. A drop of Aqua/Poly mount was added onto the hydrogels and then hydrogels were covered with a plain glass coverslip. Images were acquired with a Leica DMi8 inverted epifluorescence microscope fitted with a 12-bit grayscale CCD camera using a 40X dry objective. All images were acquired using Metamorph software.

### Peptidomimetic studies

CWHM-12 is a small molecule peptidomimetic of the amino acid motif Arg-Gly-Asp (RGD) which potently inhibits (IC_50_ < 10 nM) the binding of integrins αvβ1, αvβ3, αvβ5, αvβ6, αvβ8, and α5β1 to their respective extracellular matrix ligands, as has been described previously (Henderson et al., 2013). CWHM-96 is the R-isomer of CWWM-12 differing only in the orientation of its carboxyl (CO_2_H) group and is 100-1000-fold less potent than CWHM-12 for inhibition of these same integrins. Therefore, it was used as a highly structurally similar negative control compound. Stock solutions of 1 mM of the peptidomimetic compounds were prepared in 50% DMSO (in sterile water). PC12 and NIH 3T3 cells were seeded at 5 × 10^4^ cells/cm^2^ on SWCNTs/hydrogel, MWCNTs/hydrogel, and control PEG-RGDS substrates and cultured for 24 h. After 24 h of culture, the peptidomemetics were added at a final concentration of 10 μM directly to the cell medium and the cells were exposed to peptidomemetics for additional 24 h. The cell medium was exchanged with fresh medium and samples were imaged under an inverted fluorescent microscope (Zeiss, Axiovert 200M, Oberkochen, Germany).

### Statistical analysis

Results are reported as averages ± standard deviation. Statistical significance between multiple samples was tested by ANOVA followed by a post-hoc analysis and between two samples by Student's *t*-test (*p* < 0.05). A minimum of three samples from three independent experiments were tested per condition. For cell analysis, a minimum of 20 cells per image, from a minimum of six images were analyzed per condition.

## Results

### Cells adhered and spread onto SWCNTs and MWCNTs due to adsorption of adhesive proteins

**Figure 2:**
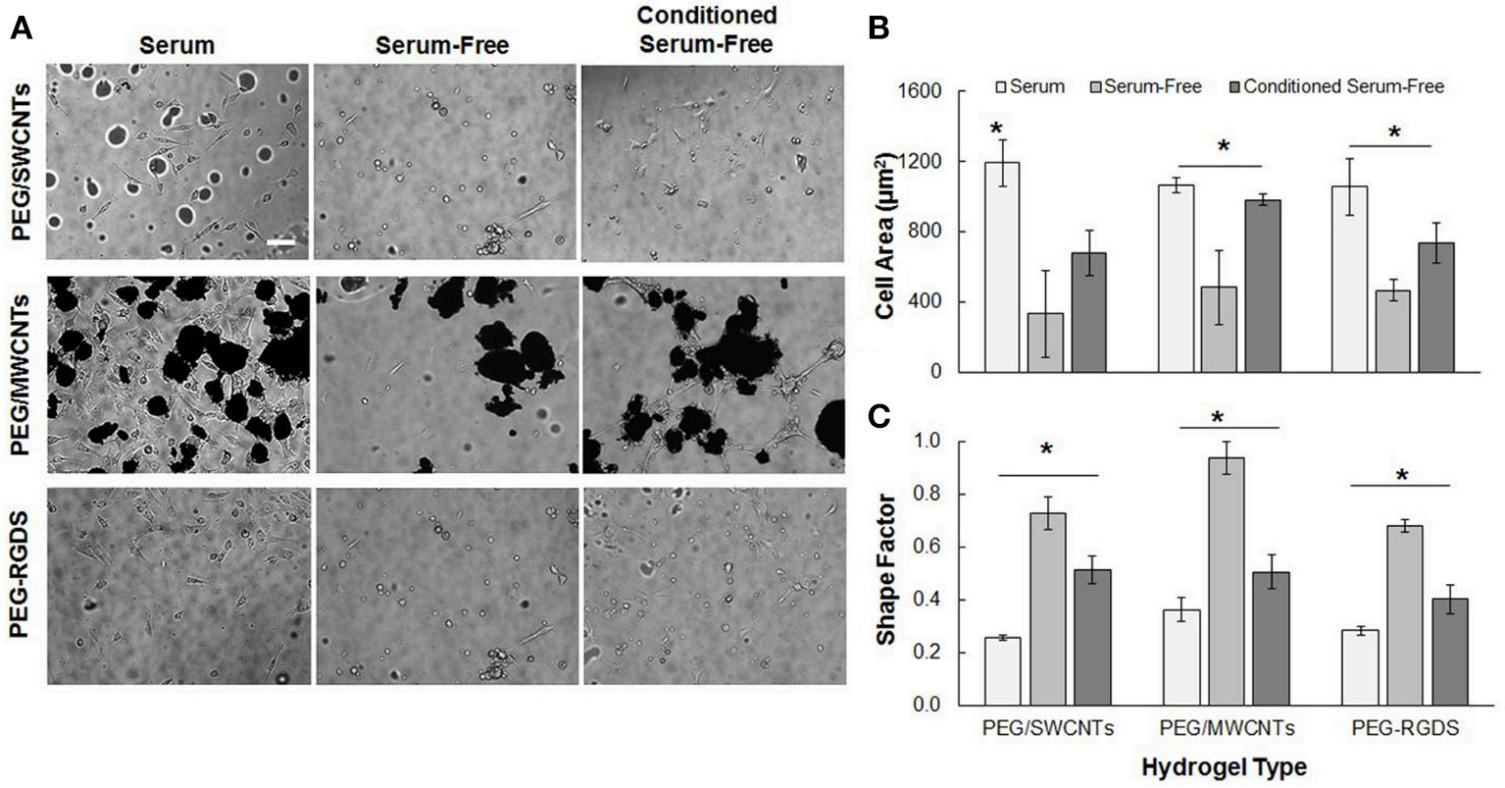
**(A)** Phase contrast images of NIH 3T3 cells on PEG/SWCNTs, PEG/MWCNTs and PEG-RGDS hydrogels in serum, serum-free and conditioned serum-free medium (scale bar is 100 μm). **(B)** Cell spreading area and **(C)** cell shape factor (inverse of elongation) for NIH 3T3 cells on PEG/SWCNTs, PEG/MWCNTs and PEG-RGDS hydrogels in serum, serum-free and conditioned serum-free medium. *Asterisks designate significant differences (120 cells from *n* = 3, *p* < 0.05).

Figure 2 shows NIH 3T3 cell attachment and spreading in serum, serum-free and conditioned serum-free medium on PEG/SWCNTs, PEG/MWCNTs, and PEG-RGDS gels. Note that PEG only hydrogel was also tested as a control, but cell attachment was not observed (data not shown). Hence, PEG-RGDS was used, where the RGDS ligand was covalently tethered to support integrin-mediated cell adhesion (Zustiak et al., 2010). To produce the conditioned serum-free medium, cells were exposed to serum-free medium for 24 h upon which the conditioned serum-free medium (i.e., containing cell-secreted biomolecules) was collected and used for experiments. Phase contrast images showed that cells adhered on all substrates independently of serum presence but exhibited serum-dependent morphology (Figure 2A). Cells had higher cell spreading area (per individual cell) and an elongated morphology (low shape factor) in the presence of serum in the medium and lower cell spreading area and a more rounded morphology (high shape factor) in the absence of serum. Quantification revealed that cell spreading area in the presence of serum was significantly higher than other media for all hydrogel types (Figure 2B). The differences were largest between the serum and serum-free media: ~56% for cells seeded on the PEG-RGDS and the PEG/MWCNTs gels and ~70% for cells seeded on the PEG/SWCNTs gels. The difference between the serum-free and conditioned serum-free medium was largest for cells seeded on the PEG/MWCNTs gels (~56%) and not significant for cells seeded on the PEG/SWCNTs gels. Shape factor was inversely proportional to cell spreading area, where lowest shape factor (i.e., highest elongation) was observed in the serum medium, followed by the conditioned serum-free and lastly by the serum-free medium (Figure 2C).

Overall, our results confirmed that serum proteins facilitated cell spreading and elongation for cells seeded on the nanocomposite hydrogels and the control PEG-RGDS gels. Conditioning the serum-free medium with cell-secreted proteins improved cell spreading and elongation over the serum-free condition but was not as effective as serum in the media. Importantly our results indicate that cells were able to adhere and spread onto unmodified SWCNTs and MWCNTs embedded in the inert PEG gels even in the absence of any adhesive ligands on the material. We suggest that the reason for cell adhesion and spreading was the ability of SWCNTs and MWCNTs to adsorb adhesive proteins (e.g., fibronectin) secreted by cells or present in serum (Khang et al., 2007), which then could elicit cell attachment and subsequent spreading.

### Exogenous and cell-secreted fibronectin adsorbed onto SWCNTs and MWCNTs; intracellular fibronectin not affected by CNTs presence

We studied the effect of fibronectin adsorption, both exogenous and cell-secreted, onto PEG/SWCNTs, and PEG/MWCNTs nanocomposite hydrogels *via* epifluorescence microscopy. To ascertain anti-fibronectin antibody specificity a negative control was used, where samples stained with normal rabbit IgG fraction were compared to samples stained with the affinity purified, rabbit anti-fibronectin antibody. Only the sample stained with the anti-fibronectin antibody showed intense TRITC fluorescence (indicated with arrows) indicating specificity (Figure S1).

**Figure 3:**
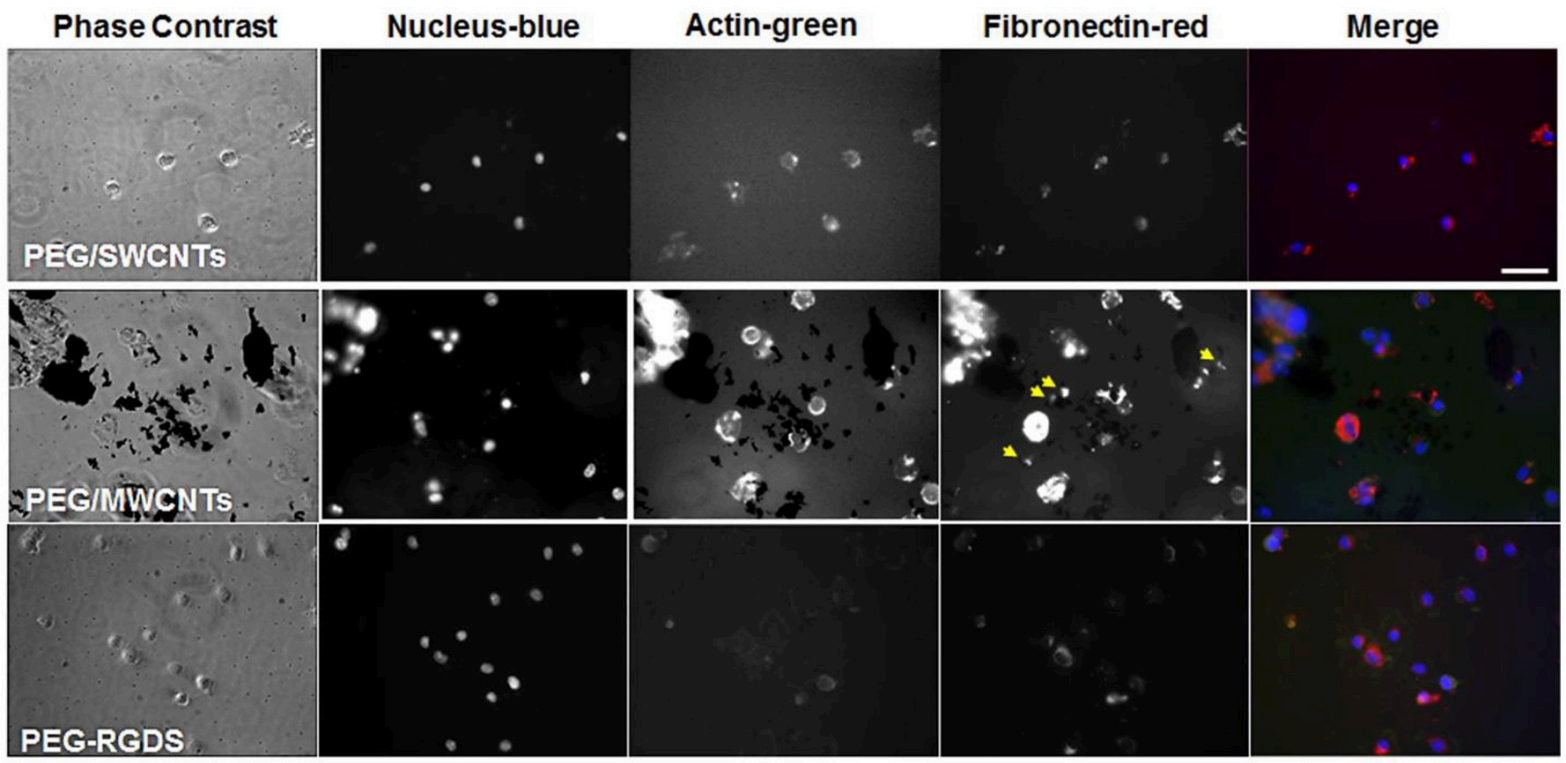
Fluorescence images of NIH 3T3 cells on PEG/SWCNTs, PEG/MWCNTs and PEG-RGDS hydrogels at 2 h in serum-free medium. Nucleus was stained with Hoechst 33258 (blue), actin was stained with Alexa 488 phalloidin (green) and fibronectin was stained with polyclonal anti-fibronectin (red). Scale bar is 50 μm. Arrows indicate extracellular fibronectin.

NIH 3T3 cells were stained for fibronectin, actin and cell nucleus to visualize intracellular and extracellular fibronectin at 2 h post-seeding in a serum-free medium for cells seeded on top of PEG/SWCNTs, PEG/MWCNTs, and PEG-RGDS gels (Figure 3). All cells stained positive for fibronectin, and there appeared to be some extracellular fibronectin at that time point, particularly on the PEG/MWCNTs nanocomposites (yellow arrows in images). At this early time point cells on all hydrogel types appeared rounded and the number of attached cells was low.

**Figure 4:**
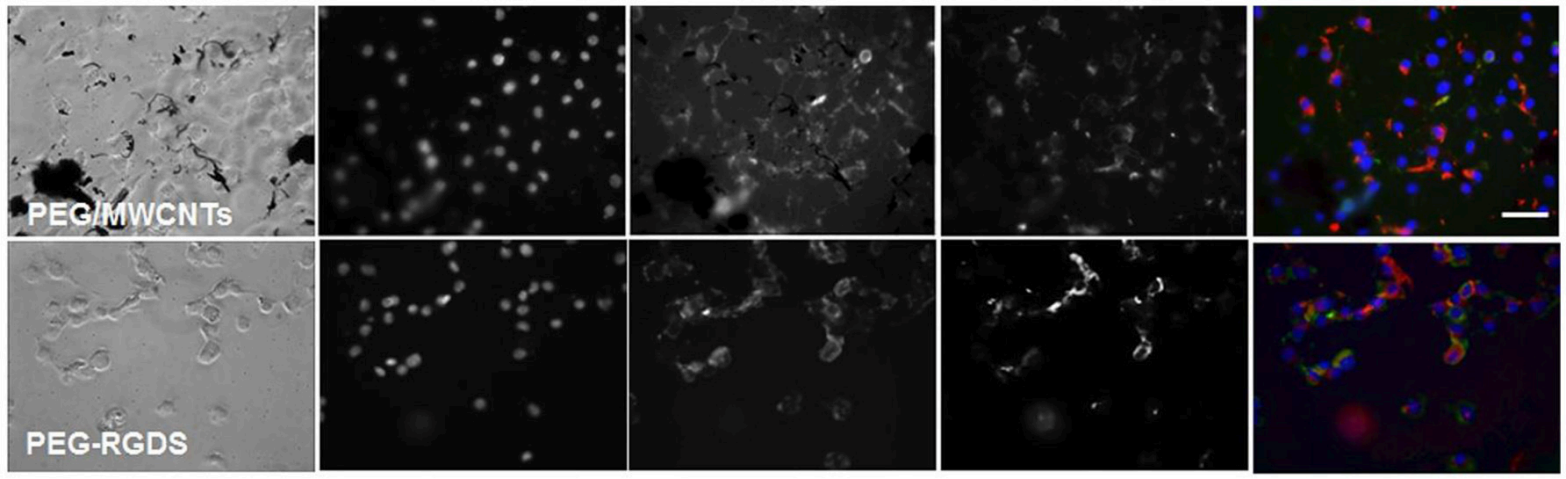
Fluorescence images of NIH 3T3 cells on fibronectin-coated PEG/MWCNTs and PEG-RGDS hydrogels at 2 h in serum-free medium. Nucleus was stained with Hoechst 33258 (blue), actin was stained with Alexa 488 phalloidin (green) and fibronectin was stained with polyclonal anti-fibronectin (red). Scale bar is 50 μm.

To determine whether pre-absorbed fibronectin will facilitate better early cell attachment and spreading, we imaged cells at 2 h of culture in serum-free medium on fibronectin-coated PEG/MWCNTs nanocomposite and the PEG-RGDS control gels (Figure 4). We specifically focused on the MWCNTs/hydrogel composite, as opposed to the SWCNTs/hydrogel one, as a representative substrate due to the easier nanotube visualization. Upon staining, we noted intracellular and some extracellular fibronectin. As expected, extracellular fibronectin was not seen on the inert PEG-RGDS gels but was seen on the PEG/MWCNTs nanocomposite due to fibronectin adsorption onto the MWCNTs. Note that we observed fibronectin only on the smaller MWCNTs clusters and not the larger ones, because we could not image the surface of the black MWCNTs with an inverted microscope. Importantly, we observed cell spreading even at this early time point and a higher number of adhered cells compared to the no-exogenous-fibronectin condition (Figure 3), especially for cells seeded on the nanocomposite hydrogel. Our results indicate that passive adsorption of adhesive proteins, such as fibronectin, facilitate cell adhesion and spreading on CNT/hydrogel nanocomposites.

Additionally, to determine whether the presence of MWCNTs affected cell production of fibronectin (i.e., intracellular fibronectin amount), we quantified the amount of fibronectin per cell. Comparing cells on the PEG/MWCNTs substrate vs. the PEG-RGDS substrate we observed no difference in intracellular fibronectin. The mean integrated pixel intensity ± s.e.m. in the PEG/MWCNT and PEG-RGDS fibronectin images was 2.7 × 10^6^ ± 0.2 × 10^6^ (*n* = 27 cells) and 3.3 × 10^6^ ± 0.3 × 10^6^ (*n* = 33 cells), respectively, yielding a *p*-value of 0.11. Hence, we conclude that MWCNT presence did not affect intracellular fibronectin levels.

**Figure 5:**
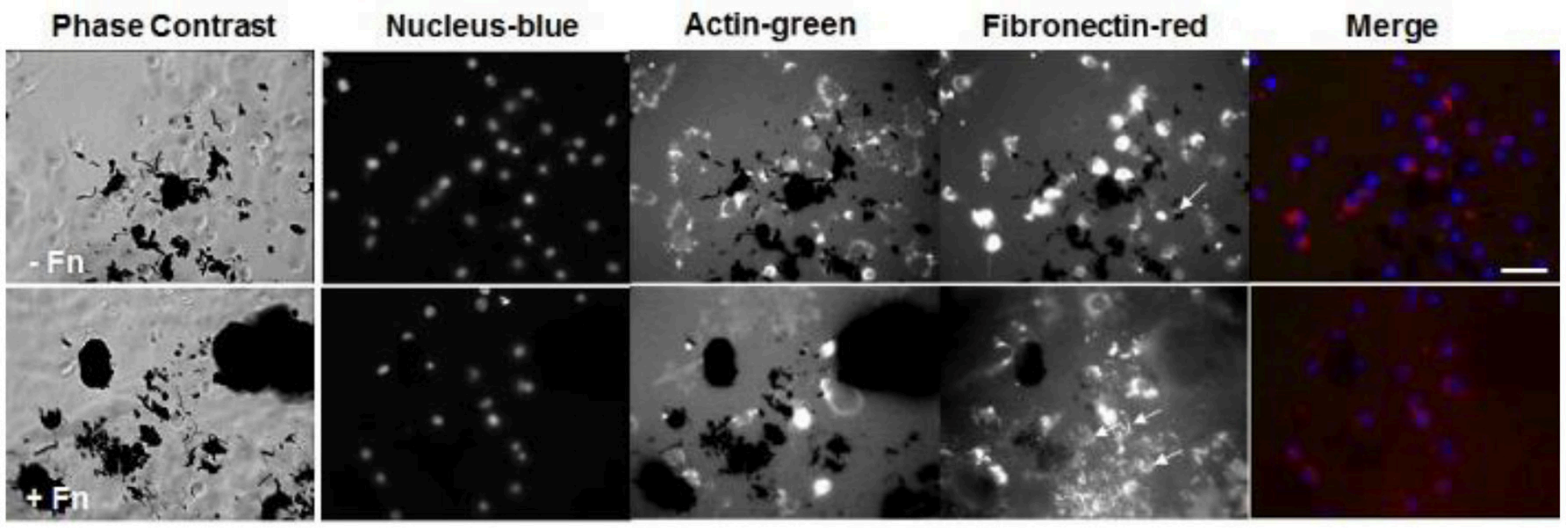
Fluorescence images of NIH 3T3 cells on PEG/MWCNTs hydrogels (−Fn) and fibronectin-coated PEG/MWCNTs hydrogels (+Fn) at 6 h in serum-free medium. Nucleus was stained with Hoechst 33258 (blue), actin was stained with Alexa 488 phalloidin (green) and fibronectin was stained with polyclonal anti-fibronectin (red). Scale bar is 50 μm. Arrows indicate extracellular fibronectin.

Lastly, we stained for fibronectin at a longer time point of 6 h for cells seeded onto PEG/MWCNTs nanocomposites expecting to see more cell-secreted extracellular fibronectin and higher number of adhered cells (Figure 5). We tested two conditions: exogenously coated fibronectin (+Fn) and no exogenous fibronectin (-Fn). We observed a similar number of attached cells irrespective of exogenous fibronectin. All cells stained positive for intracellular fibronectin; we did not note qualitative differences between cells on the fibronectin-coated samples (+Fn) and the uncoated ones (−Fn). Similarly to cell images at 2 h post-seeing, at 6 h post-seeding we observed some extracellular fibronectin in the (−Fn) condition (indicated by yellow arrows in the images), which appeared to accumulate around MWCNTs in the gel and was not seen in MWCNT-devoid regions. The fibronectin-coated samples (+Fn) exhibited an overall higher level of fluorescence from the hydrogel surface indicative of protein adsorption onto the MWCNTs. Our results indicate that cells were able to secrete fibronectin, which was subsequently adsorbed onto the MWCNTs in the nanocomposites and elicited cell adhesion and spreading. Lastly, while the addition of exogenous fibronectin led to improved cell adhesion at 2 h post-seeding (Figures 3, 4), it did not seem to affect cell adhesion at 6 h post-seeding; similar number of adhered cells were noted on both (−Fn) and (+Fn) substrates.

### Cells adhered and spread onto SWCNTs and MWCNTs on PEG hydrogels *via* integrin binding

**Figure 6:**
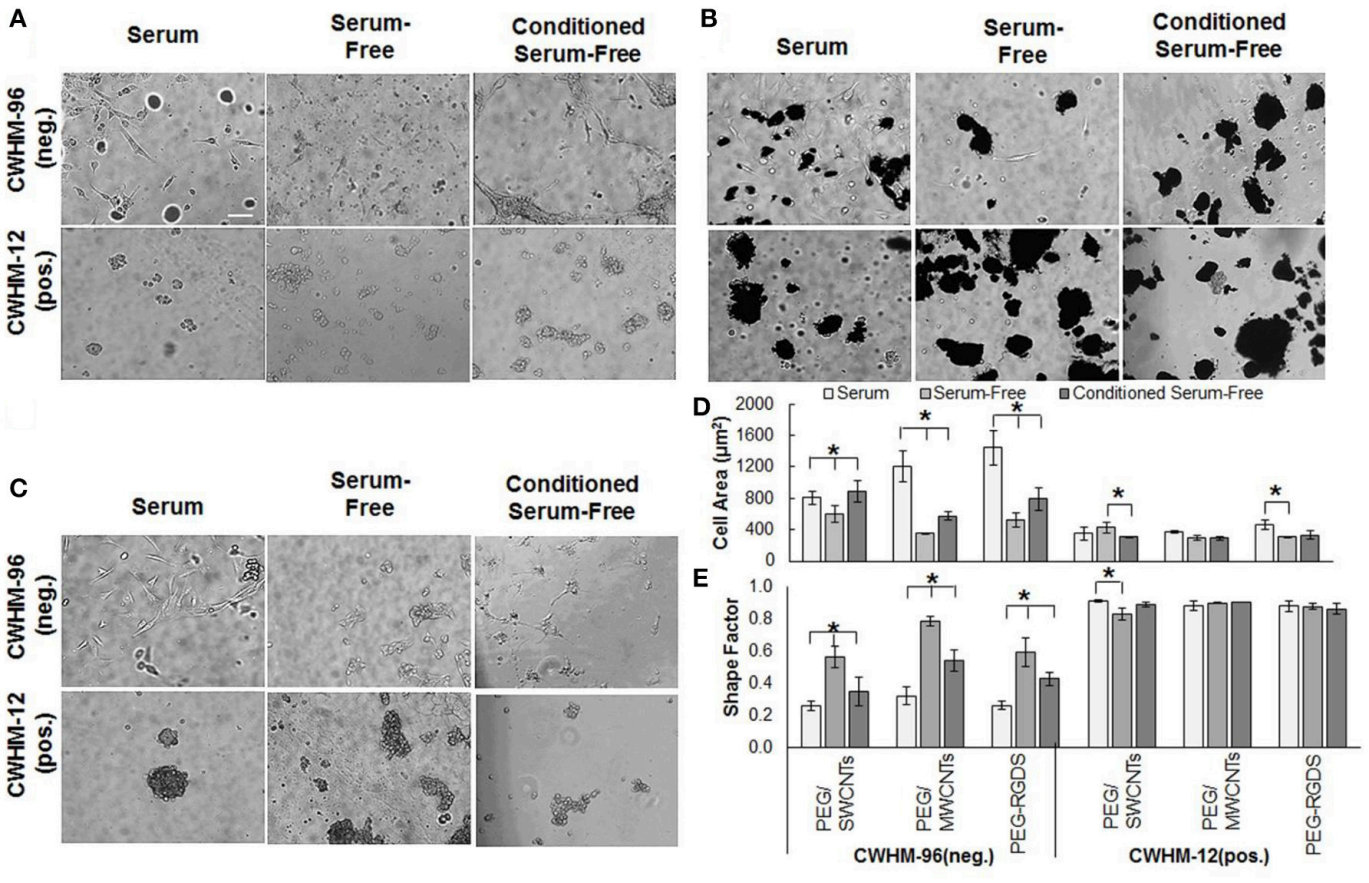
**(A)** Phase contrast images of NIH 3T3 cells in serum, **(B)** serum-free, **(C)** conditioned serum-free media on PEG/SWCNTs, PEG/MWCNTs, and PEG-RGDS hydrogels at 24 h after exposure to peptidomimetics (scale bar is 100 μm). **(D)** Cell area and **(E)** shape factor of NIH 3T3 cells on PEG/SWCNTs, PEG/MWCNTs and PEG-RGDS hydrogels in serum, serum-free and conditioned serum-free medium in the presence of CWHM-12 (active) and CWHM-96 (inactive control) peptidomimetics. *Asterisks designate significant differences (120 cells from *n* = 3, *p* < 0.05).

Next, we tested whether cell attachment and spreading onto PEG/MWCNTs and PEG/SWCNTs hydrogels was facilitated by integrin binding (Figure 6). Note that the SWCNTs in the PEG/SWCNTs hydrogels were 1–2 nm in diameter (Imaninezhad et al., 2017); hence physical entanglement of cell processes on SWCNTs alone could not explain cell attachment and spreading (Sorkin et al., 2009). We tested a highly stable synthetic RGD peptidomimetic, namely CWHM-12, with selective inhibition toward integrins α*νβ*1, α*νβ*3, α*νβ*5, α*νβ*6, α*νβ*8, and α5β1, which collectively mediate binding to RGD-containing biomolecules present in serum such as fibronectin and vitronectin. In contrast, the R-enantiomer of this compound, namely CWHM-96, has very low activity toward the same integrins.

We tested the effect of the integrin inhibitor CWHM-12 and the inactive control CWHM-96 on NIH 3T3 cell attachment and spreading onto PEG/SWCNTs, PEG/MWCNTs, and PEG-RGDS gels in serum (Figure 6A), serum-free (Figure 6B) and conditioned serum-free (Figure 6C) medium. For all conditions tested, the cells rounded up and formed cell aggregates on top of the gels when exposed to the active CWHM-12, but not when exposed to the inactive CWHM-96 compound. When quantifying cell area (Figure 6D) and shape factor (Figure 6E), we observed that both depended on the media and the substrate in the presence of the inactive compound CWHM-96, but not in the presence of the active compound CWHM-12 where all cells were rounded. As expected, in the presence of CWHM-96 cells were most spread and elongated in the presence of serum in the media, followed by conditioned serum-free, followed by serum-free medium. The differences between the three media conditions were most pronounced for the PEG-RGDS and PEG/MWCNTs gels, but were minimal for cells seeded on the PEG/SWCNTs gels. In the presence of the active compound CWHM-12 all cells had a similar cell area of ~400 μm^2^ and a shape factor close to 1, which is indicative of a circular shape, suggesting an inhibition of integrin binding.

We also tested cells seeded on PEG/SWCNTs gels in serum-free and conditioned serum-free medium after a shorter exposure of 4 h to peptidomimetics CWHM-12 and CWHM-96 (data not shown). We observed cells rounding upon exposure to the active CWHM-12 compound in both medium conditions and not in response to the inactive CWHM-96 one, similarly to data presented for 24 h of exposure. Overall, our results show that cells attached and spread *via* integrin binding onto all substrates in all media conditions, indicating that serum or cell-secreted adhesive proteins adsorb on the nanotubes and elicit cell attachment and subsequent spreading.

### Integrin-facilitated cell adhesion and spreading to SWCNTs and MWCNTs was cell-type independent

**Figure 7:**
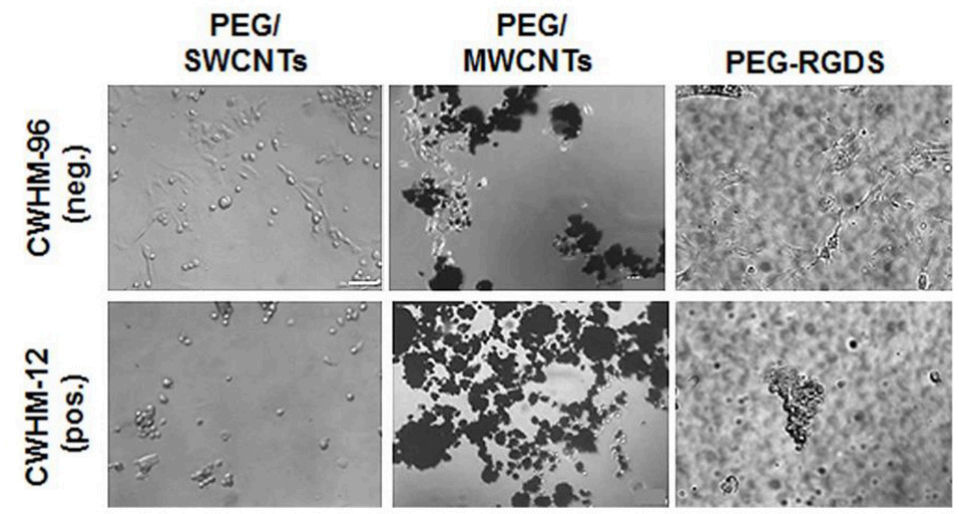
Phase contrast images of PC12 cells in serum-free medium upon exposure to CWHM-12 (pos.) and CWHM-96 (neg.) peptidomimetics. Scale bar is 100 μm.

Lastly, to confirm that cell attachment and spreading onto SWCNTs and MWCNTs was not specific to NIH 3T3 cells, we used PC12 cells in one representative experiment (Figure 7). Specifically, we seeded PC12 cells on PEG/SWCNTS, PEG/MWCNTS, and PEG-RGDS gels in serum-free medium containing inactive CWHM-96 or active CWHM-12 compounds. As with NIH 3T3 cells, PC12 cells were able to attach and spread onto all substrates in all media conditions. When exposed to the inactive CWHM-96 compound cells seemed more spread and elongated but were completely rounded in the presence of the active CWHM-12 compound. Our data indicates that PC12 cells, similarly to NIH 3T3 cells adhered and spread onto SWCNTs and MWCNTs *via* integrin binding to cell-secreted proteins adsorbed onto the nanotubes.

## Discussion

The objective of this study was to understand how cells adhere and spread onto MWCNTs aggregates and individual SWCNTs of diameter less than 2 nm embedded in an inert PEG hydrogel substrate. The significance of embedding the nanotubes in PEG substrate was to isolate cell adhesion and spreading onto the nanotubes, both SWCNTs and MWCNTs, from adhesion and spreading onto the underlying substrate: PEG is inert and does not support cell adhesion (Imaninezhad et al., 2017). We postulated that cells adhere and spread onto individual SWCNTs and MWCNTs aggregates *via* integrin binding because of serum or cell-secreted proteins adsorbed onto the CNTs' surface. Proteins are known to adsorb tightly onto the CNT surface due to pi-stacking (Salvador-Morales et al., 2006; Kaiser et al., 2013). This study was enabled by our previous work, where we developed a method to transfer individual SWCNTs onto hydrogel substrates and demonstrated robust cell adhesion to these novel nanocomposite materials (Imaninezhad et al., 2017). Because the SWCNTs were of a very small diameter (<2 nm) and dispersed individually, we postulated that physical entanglement between cellular processes and SWCNTs could not explain cell adhesion and spreading, especially because the cellular processes were of greater dimension than the individual SWCNTs; previous work has shown that neural cells can attach to MWCNT aggregates by physical entanglement of cellular processes with the nanotubes if both have similar dimensions (Sorkin et al., 2009).

To test our hypothesis, we cultured cells on PEG/SWCNTs nanocomposites, PEG/MWCNTs nanocomposites and PEG-RGDS hydrogels (no CNTs control) in various media, namely serum, serum-free, and conditioned serum-free. Note that both nanocomposite substrates, PEG/MWCNTs and PEG/SWCNTs, were developed and characterized by us previously, and have shown excellent biocompatibility with >90% cells viability retained at 24 h post-culture (Shah et al., 2015; Imaninezhad et al., 2017). The conditioned serum-free medium was used to control for the effect of cell-secreted adhesive proteins (e.g., fibronectin) as this medium was considered enriched in such proteins. The PEG-RGDS gels were used as a control substrate, since cells could only attach to the RGDS ligand in those gels. We anticipated that cell-secreted proteins will adsorb onto the CNTs and facilitate a more robust cell adhesion and spreading in this condition compared to the serum-free condition (Vogel et al., 1980). At 24 h of culture, we noted some cell adhesion in all media conditions with the highest number of cells observed in the serum, followed by the conditioned serum-free, followed by the serum-free media (Figure 2). It is important to note that cells clustered around nanotube-rich areas, which was most clearly visible on the PEG/MWCNTs substrates. As expected, the highest cell spreading was also seen in the serum medium and the lowest in the serum-free medium. Further, since fibroblasts are known to secrete adhesive proteins shortly after culturing in serum-free medium (Grinnell and Feld, 1979), our 24 h time point meant that even cells in the serum-free medium would be able to secrete proteins to facilitate their adhesion and spreading onto the surface. This result led us to believe that adsorption of adhesive proteins, either from the serum or cell-secreted, was critical and facilitated cell adhesion and spreading onto both SWCNTs and MWCNTs.

To probe for cell adhesion and spreading as well as for adhesive protein secretion at shorter time points, we stained cells cultured in a serum-free medium for 2 and 6 h for intracellular fibronectin (Figures 3–5). Fibronectins are adhesive glycoproteins on the cell surface and in the extracellular matrix and a common soluble protein secreted by cells (Akiyama and Yamada, 1985). Fibronectin has been shown to adsorb tightly and with high affinity onto carbon nanotubes (Khang et al., 2007). On the other hand, studies on the effect of soluble fibronectin binding to the surface of cells have shown that fibronectin almost exclusively binds to the external surface of the plasma membrane (Akiyama and Yamada, 1985). Fibroblast cells in particular have been shown to attach and spread in a serum-free medium because of cell-secreted fibronectin within 60 min of seeding (Grinnell and Feld, 1979).

We hypothesized that cells were able to secrete fibronectin, which would then adsorb with high affinity to the SWCNTs and MWCNTs, facilitating cell adhesion and spreading. As anticipated, at 2 h we noted that cells on all substrates stained positive for intracellular fibronectin, but we observed minimal extracellular fibronectin adsorbed on the carbon nanotube substrates (Figure 3). It could be due to the early time point, but it also could be due to the high intensity of intracellular fibronectin obscuring any weaker signal from the extracellular one. Hence, to confirm that fibronectin indeed facilitated cell adhesion, we pre-coated PEG/MWCNTs and PEG-RGDS substrates with exogenous fibronectin and cultured cells for 2 h (Figure 4). We observed a higher number of attached cells on the exogenously coated PEG/MWCNTs nanocomposites compared to the non-coated ones. We then repeated the experiment at a later time point, namely 6 h, at which point we anticipated that cells would secrete sufficient amount of fibronectin to facilitate robust cell adhesion and spreading (Grinnell and Feld, 1979) (Figure 5). At 6 h, we saw a larger number of cells adhering to the PEG/MWCNTs substrate compared to the 2 h time point in serum-free medium (48 ± 21 cells/mm^2^ vs. 27 ± 14 cells/mm^2^, respectively), where the cells were also more spread. Importantly, at 6 h there was no difference between numbers of adhered cells on the fibronectin-pre-coated vs. non-coated PEG/MWCNTs nanocomposites. Hence, our results suggested that a soluble cell-secreted adhesive protein such as fibronectin facilitated cell adhesion and spreading onto CNTs.

Finally, we did not see a significant difference in intracellular fibronectin expression for cells seeded on PEG/MWCNTs vs. PEG-RGDS substrates (Figure 4), suggesting that MWCNTs did not stimulate fibronectin expression. The effect of surface tethered MWCNTs on fibronectin expression from fibroblast cells had not been studied before. However, multiple studies have indicated that the microenvironment could affect fibronectin expression and secretion by cells. For example, it has been shown that surface roughness can increase fibronectin expression and secretion by fibroblast cells, and that fibronectin expression is greatly enhanced in high serum conditions and minimal in low serum conditions (Chou et al., 1995). Here, the PEG/MWCNTs substrate should have presented higher roughness than PEG-RGDS due to the presence of MWCNT aggregates, but the cells were cultured in the absence of serum, which could explain the similar expression levels for both conditions. However, another study on the effect of soluble SWCNTs on HEK293 cells has shown that SWCNTs down-regulated the expression of several adhesion proteins such as laminin, fibronectin, cadherin, FAK, and collagen IV (Cui et al., 2005). The difference between this finding and our results could be due to the different presentation of CNTs (soluble vs. tethered) as well as the different cell types and CNTs types used.

While we showed that cell adhesion and spreading was facilitated by adhesive proteins adsorbed onto the nanotubes, the question still remained whether the cell binding and spreading onto SWCNTs and MWCNTs was integrin-dependent. Integrins are heterodimers consisting of an α and a β subunit that together determine ligand specificity and initiate intracellular signaling events (Lowin and Straub, 2011). Fibroblast cells express the β1, β3, β5, αv, and α5 subunits of RGD-binding integrins that mediate cellular processes such as adhesion, spreading and migration (Lowin and Straub, 2011). Within the aminoacid sequences of proteins such as fibronectin and vitronectin, integrins bind to the arginine-glycine-aspartic acid (RGD) motifs (Sarin et al., 2005). Interaction with the RGD sequence is an important switch for integrin activation and subsequent cellular changes. The same ECM molecules can recognize various integrin combinations. For example, fibronectin is recognized by α4β1, α5β1, α*νβ*1, α*νβ*3, and α3β1, and weakly by others (Lowin and Straub, 2011).

Here, we used a potent small-molecule inhibitor of α5 (α5β1) and αν integrins (α*νβ*1, α*νβ*3, α*νβ*5, α*νβ*6, and α*νβ*8), namely the RGD peptidomimetic CWHM-12, and an inactive peptidomimetic CWHM-96 as control (Henderson et al., 2013). Specifically, we studied the effect of the peptidomimetics on cell attachment and spreading on PEG/SWCNTs nanocomposites, PEG/MWCNTs nanocomposites and PEG-RGDS control hydrogels in serum, serum-free and conditioned serum-free media (Figure 6). Our goal was to determine whether cell attachment and spreading onto the nanotubes in the various media conditions was dependent on RGD-binding integrins. On all substrates and in all media conditions, cells adhered and spread (higher spreading in the serum and conditioned serum-free media) in the presence of the inactive CWHM-96 compound, but balled up and formed clusters in the presence of the active CWHM-12 compound. Similar responses to the peptidomimetics were seen with another cell type, namely PC12 cells (Figure 7), indicating that the response was not cell-specific. Note that cell rounding was not caused by peptidomimetic toxicity but due to its ability to inhibit integrin binding with the substrate (Henderson et al., 2013). Hence, our results indicated that cell adhesion and spreading onto SWCNTs and MWCNTs was similar to the cell adhesion and spreading onto the control PEG-RGD gels and was integrin-dependent. While studies with RGD-like peptidomimetics have not been performed before, other studies have shown that cells are able to form focal adhesion complexes on CNTs (Ryoo et al., 2010), indicative of integrin binding.

One limitation of our study is that we did not specifically study short time points (<30 min) to determine if initial cell attachment is also dominated by integrin binding. For example, some recent work on cell adhesion to charged polymers has suggested that short term cell adhesion in the presence and absence of serum in the media is different and could not be fully explained by either integrin binding or charged interactions (Hoshiba et al., 2018). Hence, it is possible that a different and yet unidentified mechanism is guiding cell adhesion to CNTs initially, but is masked by a predominant integrin-dependent adhesion at the later time points studied here. We were also not able to unequivocally rule out that cells were able to attach directly to the CNTs. It has been suggested previously that SWCNTs can bind directly to integrins as well as to ECM proteins first, where the whole complex subsequently binds to integrins (Kaiser et al., 2013). However, it was only a suggestion based on observations by the researchers and was not proven unequivocally. Studies to answer such follow up questions will be included in our future work.

## Conclusion

In summary, we observed that NIH 3T3 cells attached and spread onto the PEG/SWCNTs and PEG/MWCNTs nanocomposites due to adhesive proteins from serum in the medium, where proteins adsorbed onto the SWCNTs and MWCNTs. Cells also adhered and spread onto the nanocomposites in serum-free medium due to cell-secreted adhesive proteins, such as fibronectin. Conditioning the serum-free medium with cell-secreted proteins enhanced cell attachment and spreading. All cells stained positive for intracellular fibronectin at 2 and 6 h of culture, where MWCNTs and SWCNTs presence did not affect intracellular fibronectin expression. Inhibition of cell attachment and spreading onto the nanocomposite materials by a broad-action RGD peptidomimetic (active against α_ν_β_1_, α_ν_β_3_, α_ν_β_5_, α_ν_β_6_, and α_ν_β_8_ integrins) confirmed that cell attachment and spreading onto the nanotubes was integrin-dependent. Similar results were observed with two different cell types, namely NIH 3T3 fibroblasts and PC12 neural-like cells, indicating that the process of cell attachment and spreading onto nanotubes was cell type-independent. This was the first study to explore why and how cells attach and spread onto both individual SWCNTs (<2 nm in diameter) and MWCNTs aggregates, embedded in an inert PEG substrate (to prevent non-specific cell attachment). Our results are significant because they demonstrated that cells used the same process for adhesion and spreading onto nanotubes and adhesive ligands such as RGDS. Importantly, we were able to show that cells recognized and bound to nanotubes which were significantly smaller than the smallest cell processes.

## Author contributions

MI conducted most of the experiments and wrote a first draft of the paper. JS performed the immunostaining work and provided associated figures and some text related to this work. DG and PR provided the peptidomimetics and contributed to experimental design related to peptidomimetics. IK assisted with SWCNT samples grown on quartz. SZ conceptualized the idea, guided experimental design and data analysis and wrote the final paper.

### Conflict of interest statement

The authors declare that the research was conducted in the absence of any commercial or financial relationships that could be construed as a potential conflict of interest.
