# Supplementary material for: Cell Attachment and Spreading on Carbon Nanotubes Is Facilitated by Integrin Binding
Source: Front Bioeng Biotechnol. 2018 Sep 24;6:129. doi: 10.3389/fbioe.2018.00129 (PMC6165858; doi:10.3389/fbioe.2018.00129)
Supplement: Supplementary file 1 [file Table_1.DOCX]

**Supplemental Data File**

**Cell Attachment and Spreading on Carbon Nanotubes is Facilitated by Integrin Binding**

Mozhdeh Imaninezhad^1^, Joseph Schober^2^, David Griggs^3^, Peter Ruminski^4’#^, Irma Kuljanishvili^5^, Silviya Petrova Zustiak^1,*^

^1^ Biomedical Engineering, Saint Louis University, Saint Louis, MO

^2^Pharmaceutical Sciences, Southern Illinois University, Edwardsville, IL

^3^Molecular Microbiology & Immunology, Saint Louis University, Saint Louis, MO

^4^Center for World Health and Medicine, Saint Louis University, Saint Louis, MO

^#^Current affiliation: Department of Medicine, Division of Oncology, Washington University, St. Louis, MO

^5^Physics, Saint Louis University, Saint Louis, MO

*To whom correspondence should be addressed:

Silviya Petrova Zustiak

Department of Biomedical Engineering

Saint Louis University

Saint Louis, MO, USA, 63103

Tel: 314-977-8331

Fax: 314-977-8403

E-mail: [silviya.zustiak@slu.edu](mailto:silviya.zustiak@slu.edu)


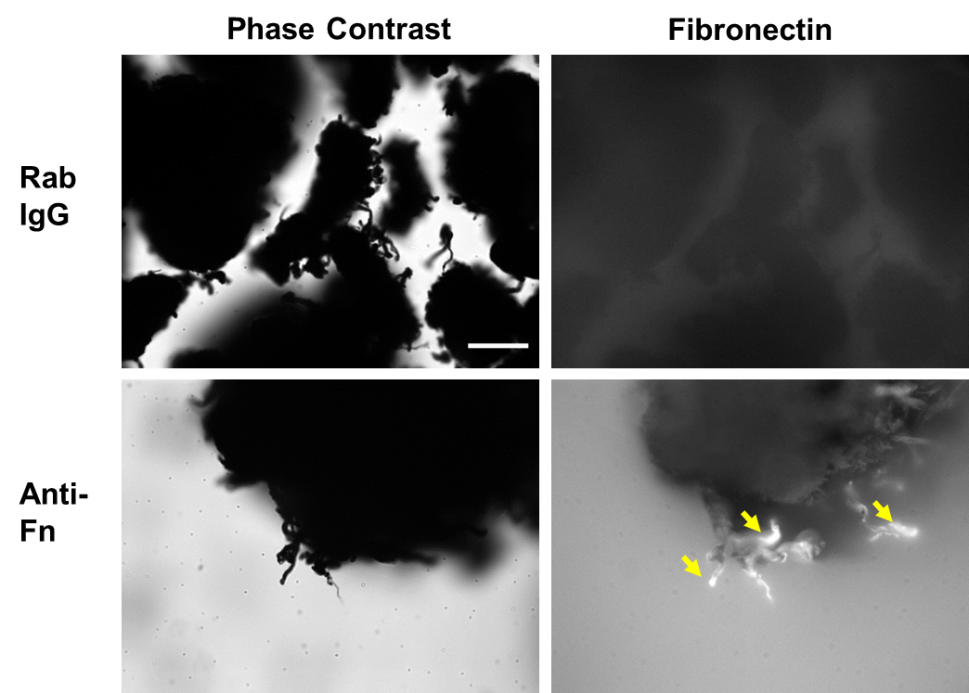


**Figure S1:** PEG/MWCNTs samples were coated with 50 μg/mL fibronectin, fixed, washed, blocked with BSA, incubated with normal Rab IgG or Rab anti-Fn antbody, washed, incubated with anti-Rab-TRITC secondary, washed and then mounted as described in Materials and Methods. Phase contrast and TRITC (Fibronectin) image channels were acquired with a 63X objective. Scale bar is 20 μm.
